# Urine Output Response to a Furosemide Infusion in Infants After Cardiopulmonary Bypass as a Predictor of Acute Kidney Injury

**DOI:** 10.1007/s00246-025-03929-y

**Published:** 2025-06-28

**Authors:** Alyson R. Pierick, Kera E. Luckritz, Ashley Huebschman, Ashley Duimstra, Sunkyung Yu, Nathaniel Sznycer-Taub

**Affiliations:** 1 Divison of Pediatric Cardiology, Department of Pediatrics, University of Michigan, Ann Arbor, MI USA; 2 Division of Pediatric Nephrology, Department of Pediatrics, University of Michigan, Ann Arbor, MI USA; 3 Department of Pediatric Pharmacy, University of Michigan, Ann Arbor, MI USA

**Keywords:** Furosemide, Thoracic surgery, Acute kidney injury, Cardiopulmonary bypass, Length of stay

## Abstract

Acute kidney injury (AKI) is a common complication in infants after cardiac surgery. Prior studies have demonstrated that urine output response to bolus dose furosemide correlates with AKI development but have excluded infants receiving a furosemide infusion. We sought to determine if urine output in response to a furosemide infusion in infants after cardiac surgery predicts AKI development. Single center retrospective cohort study of infants post cardiac surgery requiring cardiopulmonary bypass and received a post-operative furosemide infusion. A furosemide response score (FRS) (urine output [mL]/furosemide delivered [mg/kg]) was calculated. The FRS was used to determine optimal cut-offs to predict clinically significant AKI (CS-AKI), defined as stage 2 or 3 AKI. A furosemide infusion was started at a median of 9.4 h (interquartile range 6.6–13.6 h) after intensive care unit (ICU) admission in 155 infants. The post-operative incidence of AKI was 76.8%, with 44.5% having CS-AKI. The optimal FRS cut-off to correlate with AKI was 11.3 mL/mg/kg at 4 h (area under the curve [AUC] = 0.75), 25.5 mL/mg/kg at 10 h (AUC = 0.70), and 53.3 mL/mg/kg at 24 h (AUC = 0.70) post-infusion initiation, and independently associated with the development of AKI. Lower FRS also correlated with increased mechanical ventilation days and ICU/hospital length of stay. Urine output in response to a furosemide infusion in infants following cardiac surgery is associated with post-operative CS-AKI. The FRS can be used to predict AKI and potentially improve hemodynamics while minimizing risks.

**Supplementary Information:**

The online version contains supplementary material available at 10.1007/s00246-025-03929-y.

## Introduction

Acute kidney injury (AKI) following cardiac surgery in infants (< 1 year of age) is relatively common, occurring in up to 60% of cases [1–4]. Development of AKI may be related to sub-optimal renal perfusion during bypass, use of deep hypothermic circulatory arrest, inflammatory response to cardiopulmonary bypass, immature kidneys, low cardiac output post-operatively, and/or administration of blood products [4–6]. There have been many efforts to prevent AKI in the post-operative period including the use of milrinone, dexmedetomidine, corticosteroids, modified ultrafiltration, peritoneal dialysis, and remote ischemic reconditioning with no significant positive findings [7]. Development of AKI is associated with longer mechanical ventilator times, longer intensive care unit (ICU) and hospital length of stay, and long-term risk of chronic kidney disease and hypertension [6, 8–10]. Thus, finding ways to predict and prevent AKI may improve patient care and mitigate long-term complications.

Multiple studies have shown that decreased urine output in response to an intermittent dose furosemide has been shown to predict development of AKI in children following cardiopulmonary bypass [11, 12]. However, these studies have excluded patients receiving furosemide infusions post-operatively. Continuous furosemide infusions lead to higher urine output for the same total amount of furosemide received with more consistent output over time, fewer fluid bolus requirements, and improved hemodynamic stability [13–16]. Adult studies have shown furosemide infusions in the post-operative period to be safe and associated with a decreased need for renal replacement therapy [17–19]. Children who receive furosemide infusions after cardiac surgery tend to be younger, undergo more complex surgeries, and are more hemodynamically unstable. Therefore, we sought to determine if decreased urine output in response to a furosemide infusion in this population could predict development of post-operative AKI. We hypothesized that poor response to furosemide infusion in the post-operative period correlates with AKI development and would correlate with longer ventilator days and longer ICU and hospital length of stay.

## Materials and Methods

This was a retrospective, single center cohort study using routinely collected data in the electronic medical record, from January 1, 2020 through August 31, 2023. All infants less than one year of age who had cardiac surgery requiring cardiopulmonary bypass who were administered a furosemide infusion within the first 48 h post-operatively were included. Exclusion criteria included: intermittent dose of furosemide or another loop diuretic prior to infusion initiation in the post-operative period, prior need for dialysis, prematurity (< 37 weeks corrected gestational age) at the time of surgery, on extracorporeal membrane oxygenation support pre- or post-operatively, no foley catheter to aid in hourly urine output assessments, any prior surgery requiring cardiopulmonary bypass, or following heart ± kidney transplant. The standard of care for patients undergoing cardiac surgery with cardiopulmonary bypass is to receive modified ultrafiltration at the conclusion of the case. Acute kidney injury was defined using Kidney Disease: Improving Global Outcomes (KDIGO) [20–22] consensus guidelines, which has been validated in this patient population. KDIGO guidelines are dependent on deviations from baseline serum creatinine, which we defined as the lowest serum creatinine value within the preceding 6 months. Stage 1 AKI is a rise of ≥ 0.3 mg/dL within 48 h or ≥ 1.5–1.9 times the baseline creatinine, stage 2 AKI is a serum creatinine ≥ 2.0–2.9 times the baseline creatinine and stage 3 AKI is ≥ 3.0 times the baseline creatinine or receipt of dialysis. Stage 2 and 3 AKI were defined as clinically significant AKI (CS-AKI) throughout the study. Exposure to nephrotoxic agents was based on previously published Nephrotoxic Injury Negated by Just-in-Time Action (NINJA) criteria [23]. This study was approved by the University of Michigan Institutional Review Board (IRB) and individual patient consent was not required. The procedures followed were in accordance with the ethical standards of the institutional committee on human experimentation and with the Helsinki Declaration of 1975, as most recently amended.

Demographic, pre-operative, and operative characteristics were collected through the electronic medical record. Surgical complexity was classified using the Society of Thoracic Surgeons-European Association for Cardio-Thoracic Surgery (STAT) score, with STAT category 4 and 5 considered high-risk surgeries. Vasoactive-Ionotropic Score (VIS) was determined pre-operatively if the patient was inpatient, then at 6-, 12-, 18-, 24-, and 48-h post-operatively. Serum creatinine was collected at baseline (lowest value in preceding 6 months), pre- and post-operative, and daily for the first 7 post-operative days and used to determine presence and staging of AKI. Corrected serum creatinine was calculated using the following equation: measured serum creatinine × [1 + (accumulated net fluid balance/0.6 × weight (kg))] (all data reported in supplemental tables). Other diuretics received and fluid balance were determined based on patient dosing weight for each post-operative day. Furosemide infusion start time and dose was recorded, as well as change in the infusion over the first 7 days after surgery; urine output was also recorded at each time point. The pharmacokinetics of a furosemide infusion indicate that a threshold dose of furosemide should be reached within the first couple of hours (reaches peak effect at 1–3 h in neonates and infants), and patients should reach steady state by 10 h post-initiation [24]. To account for urine output in response to administered furosemide (including changes in infusion dosing), we calculated a furosemide response score (FRS) at three times point: 4- 10- and 24-h post-infusion initiation. The FRS was developed by this team to have a standardized scoring system. The score was calculated by dividing the total urine output over each time by the total amount of furosemide received corrected for dosing weight, creating an FRS score in units of mL/mg/kg. Total mechanical ventilation days, ICU, and hospital length of stay was determined for each patient after the surgery (excluding pre-operative days).

### Statistical Analysis

Data are presented as frequency with percentage (%) for categorical variables and mean ± standard deviation or median with interquartile range (IQR) for continuous variables. Univariate comparisons of demographic, pre-operative, and operative characteristics as well as FRS at each time point and post-operative clinical outcomes were made between patients who have developed CS-AKI vs those who had no AKI or stage 1 AKI, using Chi-square test or Fisher’s exact test for categorical variables and two-sample t-test or Wilcoxon rank sum test for continuous variables. Similarly, univariate associations of demographic, pre-operative, and operative characteristics with FRS at each time point were examined using Wilcoxon rank sum test for categorical variables and Spearman correlation coefficient for continuous variables. Using receiver operating characteristic (ROC) curve, the optimal cut-off of FRS at each time point to predict the development of clinically significant AKI was determined based upon the best combination of sensitivity and specificity from the ROC curve. The area under the curve (AUC), sensitivity, specificity, positive predictive value (PPV), and negative predictive value (NPV) for each cut-off value were reported. Variables found to be significantly associated with CS-AKI or FRS in the univariate analysis (*p* < 0.05) were investigated further by building multivariable logistic regression to determine independent associations of the optimal cut-off of FRS at each time point with CS-AKI. Variance inflation factor (VIF) was used to examine multicollinearity among the optimal cut-off of FRS at each time point and the candidate variables to be included in the multivariable analysis. Adjusted odds ratios with 95% confidence intervals (CIs) from multivariable logistic regression were reported. Lastly, univariate associations of the optimal cut-off of FRS at each time point with post-operative clinical outcomes were also evaluated using Fisher’s exact test for categorical variables and Wilcoxon rank sum test for continuous variables. All analyses were performed with SAS Version 9.4 (SAS institute Inc., Cary, NC), with statistical significance set at a *p*-value < 0.05 using two-sided tests.

## Results

### Patient and Surgical Characteristics

A total of 155 patients were included in the study. The majority were male (64.5%) and Caucasian (78.7%). The mean birthweight was 3.1 ± 0.62 kg with a median gestational age of 39 weeks (IQR 38–39). The most common cardiac diagnoses were d-transposition of the great arteries (18.1%), Tetralogy of Fallot (14.2%), coarctation of the aorta (10.3%), ventricular septal defect (10.3%), and hypoplastic left heart syndrome (9.7%). A total of 26 (16.8%) patients were classified as single ventricle patients and 7 (4.5%) had an underlying genitourinary abnormality. The median age of patients at surgery was 9 days old (IQR 5–96 days) with 61.3% being less than 30 days old. Over half of patients (61.9%) had a STAT category 4 or 5 surgery. No patients required dialysis in the post-operative period nor met NINJA criteria for nephrotoxic medication exposure.

Using uncorrected serum creatinine to define AKI, 50 (32.3%) patients had stage 1 AKI, 46 (29.7%) had stage 2 AKI, 23 (14.8%) had stage 3 AKI, and 36 (23.2%) had no AKI. Table 1 shows the breakdown of demographic, pre-operative, and operative characteristics for the entire cohort and comparing those with no AKI/AKI Stage 1 and CS-AKI. Infants with CS-AKI had lower birth weight and length, smaller size at surgery, more STAT category 4 or 5 surgeries, and were less likely to be on diuretics pre-operatively. This same data was analyzed using corrected serum creatinine, with few significant differences from uncorrected serum creatinine (Supplemental Table 1).

**Table 1:** Demographic, pre-operative, and operative characteristics (*N* = 155). Baseline patient cohort demographic, pre-operative, and operative characteristics and comparison based on clinically significant acute kidney injury

Characteristics	All (*N* = 155)	No AKI or AKI Stage 1 (*N* = 86)	AKI Stage 2 or 3 (*N* = 69)	*P*-value^§^
Male sex	100 (64.5)	58 (67.4)	42 (60.9)	0.40
Caucasian race	122 (78.7)	69 (80.2)	53 (76.8)	0.51
Hispanic ethnicity	10 (6.5)	7 (8.1)	3 (4.3)	0.51
Birth weight, kg (*N* = 154)	3.1 ± 0.6	3.2 ± 0.6	3.0 ± 0.6	0.02
< 2.5 kg	23/154 (14.9)	11/85 (12.9)	12/69 (17.4)	0.44
Birth length, cm (*N* = 150)	49.0 ± 3.6	49.5 ± 3.5	48.3 ± 3.8	0.04
Gestational age at birth, week	39 (38–39)	39 (38–39)	39 (37–39)	0.51
Single ventricle	26 (16.8)	14 (16.3)	12 (17.4)	0.85
Genitourinary abnormality	7 (4.5)	3 (3.5)	4 (5.8)	0.70
Pre-operative ventilation	33 (21.3)	17 (19.8)	16 (23.2)	0.61
On diuretics day prior to surgery	85 (54.8)	58 (67.4)	27 (39.1)	0.0004
PO	35 (22.6)	26 (30.2)	9 (13.0)	
IV	50 (32.3)	32 (37.2)	18 (26.1)	
Pre-operative feeding	119 (76.8)	70 (81.4)	49 (71.0)	0.13
Age at surgery, days	9 (5–96)	10 (5–102)	8 (5–50)	0.36
< 30 days	95 (61.3)	49 (57.0)	46 (66.7)	0.22
Weight at surgery, kg	4.0 ± 1.3	4.3 ± 1.3	3.7 ± 1.2	0.003
Length at surgery, cm	53.1 ± 6.0	54.2 ± 6.0	51.7 ± 5.7	0.01
BSA at surgery, m^2^	0.24 ± 0.05	0.26 ± 0.05	0.23 ± 0.05	0.003
STAT category				0.01
1 to 3	59 (38.1)	40 (46.5)	19 (27.5)	
4 or 5	96 (61.9)	46 (53.5)	50 (72.5)	
On inotropes before surgery	34 (21.9)	18 (20.9)	16 (23.2)	0.74
Milrinone	23 (14.8)	14 (16.3)	9 (13.0)	
Other	15 (9.7)	7 (8.1)	8 (11.6)	
CPB time, minutes	118 (84–150)	120 (92–150)	108 (78–149)	0.15
Aortic Cross-clamp time, minutes (n = 141)	60 (40–90)	76.5 (50–95)	54 (41–90)	0.06
DHCA	59 (38.1)	30 (34.9)	29 (42.0)	0.36
DHCA time, minutes (N = 59)	39 (30–47)	38.5 (24–43)	41 (34–48)	0.22

*AKI* acute kidney injury, *BSA* body surface area, *STAT* Society of Thoracic Surgeons-European Association for Cardio-Thoracic Surgery, *CPB* cardiopulmonary bypass, *DHCA* deep hypothermic circulatory arrest

^*^Data are presented as *N* (%) for categorical variables and Mean ± standard deviation or Median (interquartile range) for continuous variables

^§^P-value from Chi-square test or Fisher’s exact test for categorical variables and two-sample t-test or Wilcoxon rank sum test for continuous variables

### Furosemide Response and the Furosemide Response Score

**Fig. 1:**
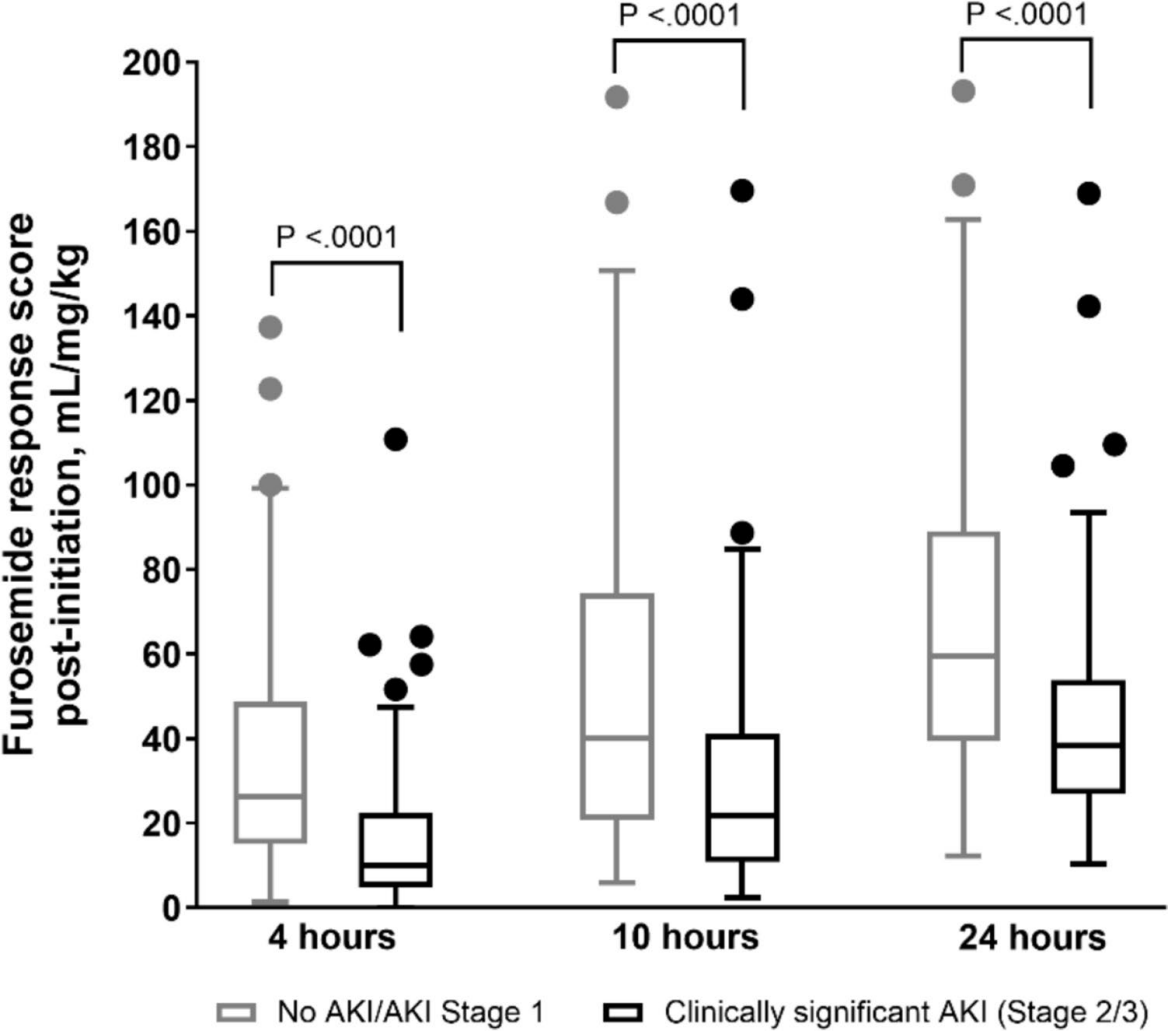
Box and whisker diagrams of furosemide response score based on development of clinically significant acute kidney injury at each time point studied

A furosemide infusion was started at a median of 9.4 h (IQR 6.6–13.6 h) after ICU admission, with the most common starting dose of 0.3 mg/kg/hr. The median amount of furosemide delivered at 4-, 10-, and 24-h post-initiation was 1.2 mg/kg (IQR 0.9–1.2), 3.0 mg/kg (IQR 2.6–3.6), and 8.1 mg/kg (IQR 6.7–9.6). Median urine output at 4-, 10-, and 24-h post-initiation was 4.7 mL/kg (IQR 2.7–8.6), 25.6 mL/kg (IQR 12.9–41.2), and 108 mL/kg (IQR 80–133). This led to a median furosemide response score (mL/mg/kg) of 18.8 (IQR 8.3–36.7), 33.1 (IQR 16.1–54.4), and 50.0 (IQR 32.6–73.9) at each time point, respectively, shown in Fig. 1. Additional diuretics received at these time points and for the first 7 post-operative days as well as overall fluid balance is shown in Supplemental Table 2. Few patients were started on additional diuretics prior to post-operative day 2, typically with chlorothiazide to augment diuresis. Patients started conversion to either intermittent intravenous or enteral furosemide on post-operative day 2.

Univariate associations of demographic, pre-operative, and operative characteristics with FRS at each time point are presented in Supplemental Table 3. Younger age, lower weight, length, and body surface area (BSA) at surgery, STAT category 4 or 5 surgery, and longer DHCA time were significantly associated with lower FRS at the three time points.

In univariate analysis, lower FRS at each time point was significantly correlated with CS-AKI (all *p* < 0.0001). Infants with development of CS-AKI had longer initial mechanical ventilation days (median 4 vs 3, p = 0.04) and ICU total length of stay days (median 8 vs 6, *p* = 0.04) compared to those with no AKI/AKI Stage 1. There was only one in-hospital death and 8 overall in the cohort, all occurring in patients with CS-AKI. CS-AKI was not correlated with VIS at any time point (all *p* > 0.20), need for additional chest tube (21.7% vs 29.1%, *p* = 0.30), delayed sternal closure (34.8% vs 24.4%, *p* = 0.16), or hospital length of stay (median 20 vs 14.5 days, *p* = 0.07).

**Fig. 2:**
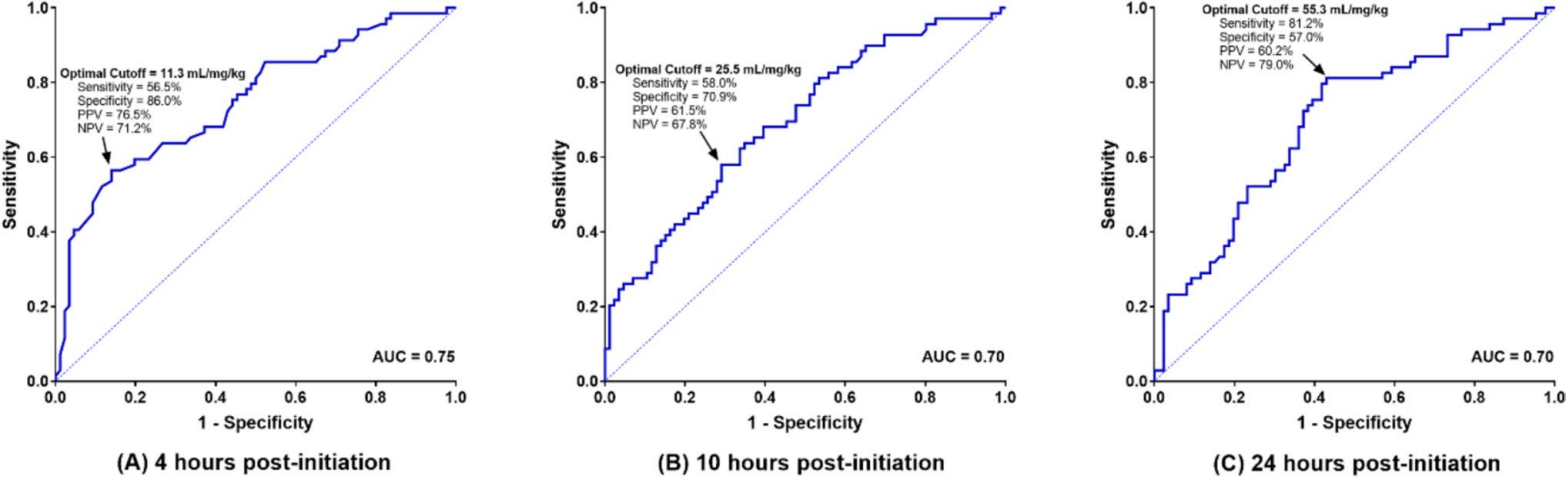
Receiver operating curves for furosemide response score prediction of clinically significant acute kidney injury at each time point studied. At 4 h: AUC = 0.75, sensitivity = 56.5% and specificity = 86.0%, PPV = 76.5% and NPV = 71.2%. At 10 h: AUC = 0.70, sensitivity = 58.0% and specificity = 70.9%, PPV = 61.5% and NPV = 67.8%. At 24 h: AUC = 0.70, sensitivity = 81.2% and specificity = 57.0%, PPV = 60.2% and NPV = 79.0%

Figure 2 depicts the ROC curves used to determine the optimal FRS cut-off at each time point with the best combination of sensitivity and specificity, which were 11.3 mL/mg/kg (AUC 0.75), 25.5 mL/mg/kg (AUC 0.70), and 55.3 mL/mg/kg (AUC 0.70), respectively.

Controlling for pre-operative feeding, BSA at surgery, STAT category 4 or 5 surgery, and ionotropic support at surgery, the FRS below the cut-offs at each time point were independently associated with CS-AKI, with an adjusted odds ratio of 6.8 (95% CI 2.9–16.1, p < 0.0001), 2.5 (95% CI 1.2–5.2, p = 0.02), and 5.3 (95% CI 2.3–11.9, p < 0.0001), respectively. The FRSs below the cut-offs were also correlated with longer mechanical ventilation days, ICU length of stay, and hospital length of stay at nearly every time point (Table 2).

**Table 2:** Univariate associations of furosemide response score (mL/mg/kg) using cut-offs from ROC curve analysis for post-operative AKI using uncorrected serum creatinine as outcome with post-operative outcomes (*N* = 155)

	4 h post-initiation			
Post-operative outcomes	< 11.3 (*N* = 51)	≥ 11.3 (*N* = 104)		*P*-value^§^
Total duration of intubation post-surgery, days	4 (3–8)	3 (2–6)		0.01
Total ICU LOS post-surgery, days	10 (5–17)	6.5 (4–11.5)		0.02
Hospital length of stay post-surgery, days	23 (13–32)	13 (9–24.5)		0.004
Deceased	4 (7.8)	4 (3.8)		0.44

	10 h post-initiation			
	< 25.5 (*N* = 65)	≥ 25.5 (*N* = 90)		*P*-value^§^
Total duration of intubation post-surgery, days	4 (3–9)	3 (2–6)		0.06
Total ICU LOS post-surgery, days	9 (5–17)	7 (4–11)		0.03
Hospital length of stay post-surgery, days	21 (12–30)	13.5 (9–24)		0.01
Deceased	5 (7.7)	3 (3.3)		0.28

	24 h post-initiation			
	< 55.3 (*N* = 93)	≥ 55.3 (*N* = 62)		*P*-value^§^
Total duration of intubation post-surgery, days	4 (3–7)	3 (2–5)		0.01
Total ICU LOS post-surgery, days	9 (5–17)	6 (4–9)		0.003
Hospital length of stay post-surgery, days	19 (11–30)	13 (9–23)		0.01
Deceased	6 (6.5)	2 (3.2)		0.48

*ROC* receiver operating curve, *AKI* acute kidney injury, *ICU* intensive care unit

^*^Data are presented as *N* (%) for categorical variables and Median (interquartile range) for continuous variables

^§^P-value from Fisher’s exact test for categorical variables and Wilcoxon rank sum test for continuous variables

## Discussion

To the authors’ knowledge, this is the first study assessing whether urine output response after furosemide infusion initiation is associated with AKI development following cardiac surgery in infants. In this cohort, FRS at 4-, 10-, and 24-h post-furosemide infusion initiation of < 11.3 mL/mg/kg, < 25.5 mL/mg/kg, and < 55.3 mL/mg/kg, respectively, was significantly associated with CS-AKI. This held true even when controlling for significant variables also associated with AKI. The FRS cut-offs at these time points were also associated with longer mechanical ventilation days and longer ICU and hospital lengths of stay. Similar findings have been shown in infants and children receiving intermittent bolus doses of furosemide [11, 12].

Our study cohort represented a patient population that was overall young at surgery (more than half were < 30 days at time of surgery) and who underwent high complexity surgeries (> 50% of the surgeries were STAT category 4 or 5). These are known risk factors for development of AKI. The overall cohort had an AKI incidence of 76.8% using uncorrected serum creatinine, which is similar to a study of neonates (< 30 days) after cardiac surgery which showed an AKI incidence of 70% [21]. We chose stage 2 or 3 AKI as clinically significant based on the fact that Taylor et al. demonstrated that stage 1 AKI in the post-operative setting was not clinically significant and did not correlate with significant clinical or long-term outcomes [25]. A few studies have assessed defining AKI based on corrected serum creatinine based on fluid overload [12, 26, 27], but there remains no consensus regarding the best creatinine to use in defining AKI. Given that we propose the FRS to be used at the bedside in real-time, we elected to present data using uncorrected serum creatinine to define CS-AKI. Our findings using corrected serum creatinine are shown in Supplemental Fig. 3 and have few differences from the data presented here.

Prior studies investigating urine output after bolus dose diuretics have simply calculated the urine output at various times after the bolus dose was administered. Given that our study question involved an infusion that could be titrated over time, we sought to use a metric that incorporated urine output in combination with the total dose of furosemide administered over time. The FRS was created in this study to be used as a clinical tool to predict clinically significant AKI. With the highest AUC, an FRS score may be calculated 4 h after furosemide infusion initiation to predict the development of AKI. We proposed that this score could be used to minimize administration of nephrotoxic agents, facilitate early discussions with nephrology, and set expectations for mechanical ventilation days and ICU/hospital lengths of stay with providers and families. Although CS-AKI at this time point likely reflects injury that has already happened, avoiding further injury can only help long-term renal function. In addition, the score could be utilized as a metric in future studies to assess the impact of pre-, intra- and post-operative interventions to reduce AKI.

Our study had a couple of surprising findings. In our cohort, infants on pre-operative diuretics were at decreased risk of developing CS-AKI. This is a rather novel findings as adult studies have shown pre-operative diuretics lead to increased risk of AKI following cardiac surgery [28]. One single-center study has shown no association with pre-operative diuretics and post-operative AKI in a similar patient population [29]. In our study, most of the patients on pre-operative diuretics were on enteral dosing, likely related to those infants who were less ill pre-operatively and at home prior to surgery. We propose that these infants were less sick going into surgery and thus had easier recovery and less likely to have AKI. Second, there was a trend toward those who had longer aortic cross-clamp time on cardiopulmonary bypass being less likely to develop clinically significant CS-AKI. This contrasts with multiple prior studies which have shown a direct correlation between cross-clamp time and AKI. We propose that this finding is likely due to multiple comparison bias or potentially related to the fact that there is not always a direct correlation between cross-clamp times and the surgical complexity or post-operative hemodynamic instability.

This study has a few important limitations. First, this was a retrospective cohort study with the inherent limitations of a retrospective study. Second, this was a single-center study allowing for consistency in care but potentially limiting its generalizability to other centers. Third, given that we excluded patients on pre- or post-operative ECMO or after heart transplantation, the results may not be applicable to these high-risk patients who may require a more targeted approach. We also included patients with underlying genitourinary anomalies, which were in 7 patients overall and mostly structural kidney diseases such as urinary tract dilation that we expected to have little effect on our outcome measures. Given that the median age of patients in our study was < 2 weeks old, pre-operative serum creatinine levels may have been elevated reflecting maternal serum creatinine, and the degree of AKI may have been underestimated for some neonates. In addition, the KDIGO guidelines also define AKI using urine output, which we chose to leave out of our definition given that this was the exploratory variable in our study.

Acute kidney injury is common following infant cardiac surgery and early identification of those at risk may help mitigate further risk and provide better counseling for families in the short- and long-term. The furosemide response score may be a helpful tool at the bedside to predict AKI after initiation of a furosemide infusion. This score could be automatically incorporated in the electronic health record for ease of use. Identifying those at risk is important, but continued work to find methods to prevent AKI is equally important.

## Supplementary Information

Below is the link to the electronic supplementary material.

Supplementary file1 (PDF 257 kb)

## Data Availability

No datasets were generated or analysed during the current study.
