# Supplementary material for: Urine Output Response to a Furosemide Infusion in Infants After Cardiopulmonary Bypass as a Predictor of Acute Kidney Injury
Source: Pediatr Cardiol. 2025 Jun 28;47(4):1520–7. doi: 10.1007/s00246-025-03929-y (PMC12945940; doi:10.1007/s00246-025-03929-y)
Supplement: Supplementary file 1 — Supplementary file1 (PDF 257 kb) [file 246_2025_3929_MOESM1_ESM.pdf]

Urine Output Response to a Furosemide Infusion in Infants After Cardiopulmonary Bypass as a Predictor of Acute Kidney Injury

Pediatric Cardiology

Authors: Alyson R Pierick, MD[1]; Kera E Luckritz, DO[2]; Ashley Huebschman, PharmD[3]; Ashley Duimstra, MS[1]; Sunkyung Yu, MS[1]; Nathaniel Sznycer-Taub, MD[1]

Corresponding Author:

Alyson R Pierick

Alysonpierick8@gmail.com

Supplemental Table 1. Univariate Associations of Demographic, Pre-operative, and Operative characteristics with Post-operative AKI (N=155)

Characteristics	Corrected Serum Creatinine: No AKI or AKI Stage 1 (N=80)	Corrected Serum Creatinine: AKI Stage 2 or 3 (N=75)	P-value[§]
Male sex	54 (67.5)	46 (61.3)	0.42
Caucasian race	63 (78.8)	59 (78.7)	0.87
Hispanic ethnicity	5 (6.3)	5 (6.7)	1.00
Birth weight, kg (N=154)	3.1 ± 0.70	3.0 ± 0.52	0.22
< 2.5 kg	13/80 (16.3)	10/74 (13.5)	0.63
Birth length, cm (N=150)	49.4 ± 4.1	48.6 ± 3.0	0.19
Gestational age at birth, week	39 (38-39)	39 (37-39)	0.55
Single ventricle	11 (13.8)	15 (20.0)	0.30
Genitourinary abnormality	3 (3.8)	4 (5.3)	0.71
Pre-operative ventilation	14 (17.5)	19 (25.3)	0.23
On diuretics day prior to surgery	55 (68.8)	30 (40.0)	0.0003
PO	28 (35.0)	7 (9.3)	
IV	27 (33.8)	23 (30.7)	
Pre-operative feeding	66 (82.5)	53 (70.7)	0.08
Age at surgery, days	28 (5.5-136)	7 (4-37)	0.02
< 30 days	40 (50.0)	55 (73.3)	0.003
Weight at surgery, kg	4.4 ± 1.4	3.6 ± 1.1	<0.001
Length at surgery, cm	54.9 ± 6.3	51.2 ± 5.0	<0.001
BSA at surgery, m^2	0.26 ± 0.05	0.23 ± 0.04	<0.001

STAT category			0.01
1 to 3	38 (47.5)	21 (28.0)	
4 or 5	42 (52.5)	54 (72.0)	
On inotropes before surgery	13 (16.3)	21 (28.0)	0.08
Milrinone	10 (12.5)	13 (17.3)	
Other	5 (6.3)	10 (13.3)	
CPB time, minutes	118 (89.5-149)	118 (80-152)	0.78
Cross-clamp time, minutes	63.5 (43.5-87.5)	54 (37-92)	0.18
DHCA	38 (33.8)	32 (42.7)	0.25
DHCA time, minutes (N=59)	38 (24-41)	41 (34-48.5)	0.12

Abbreviations: BSA, body surface area; STAT, Society of Thoracic Surgeons-European Association for Cardio-Thoracic Surgery; CPB, cardiopulmonary bypass; DHCA, deep hypothermic circulatory arrest.

* Data are presented as N (%) for categorical variables and Mean ± standard deviation or Median (interquartile range) for continuous variables.

§ P-value from Chi-square test or Fisher's exact test for categorical variables and two-sample t-test or Wilcoxon rank sum test for continuous variables.

Supplemental Table 2. Additional diuretics received after furosemide infusion initiation and overall fluid balance (N=155)									
	4 hours	10 hours	24 hours	POD2	POD3	POD4	POD5	POD6	POD7
Additional diuretics, N (%)									
None	155 (100.0)	140 (90.3)	104 (67.1)	75 (48.4)	53 (34.2)	31 (20.0)	19 (12.3)	16 (10.3)	13 (8.4)
Chlorothiazide	---	15 (9.7)	51 (21.9)	73 (47.1)	60 (38.7)	59 (38.1)	57 (36.8)	49 (31.6)	46 (29.7)
Spironolactone	---	---	---	4 (2.6)	11 (7.1)	15 (9.7)	17 (11.0)	20 (12.9)	24 (15.5)
Intravenous Furosemide	---	---	---	12 (7.7)	54 (34.8)	70 (45.2)	70 (45.2)	63 (40.6)	53 (34.2)
Oral Furosemide	---	---	---	---	5 (3.2)	28 (18.1)	45 (29.0)	64 (41.3)	71 (45.8)
Bumetanide	---	---	---	---	---	---	---	---	1 (0.6)
N/A (patient was discharged already)	---	---	--	---	---	---	1 (0.6)	1 (0.6)	6 (3.9)
Total daily fluid balance, mL									
Number	---	---	155	155	155	154	152	148	138
Mean ± SD	---	---	-31.0 ± 187	-162 ± 182	-90.7 ± 159	19.3 ± 172	47.1 ± 154	74.4 ± 141	92.9 ± 144
Range (min-max)	---	---	-517 – 438	-648 – 371	-589 – 322	-641 – 586	-326 – 692	-362 – 412	-313 – 458

*Data are presented as N (%) for categorical variables and Mean ± standard deviation and range (min-Max) for continuous variables.

Supplemental Table 3. Univariate Associations of Demographic, Pre-operative, and Operative characteristics with Furosemide response score (N=155)

Characteristics		4 hours post-initiation		10 hours post-initiation		24 hours post-initiation	
		Median (IQR) or *r*	P-value§	Median (IQR) or *r*	P-value§	Median (IQR) or *r*	P-value§
Gender			0.07		0.39		0.74
Male		20.0 (11.3-39.0)		34.0 (15.9-58.6)		51.6 (32.5-75.2)	
Female		13.8 (6.7-34.2)		29.2 (16.1-46.7)		45.2 (33.4-73.1)	
Race			0.71		0.46		0.09
Caucasian		18.8 (9.3-33.8)		32.5 (16.1-52.7)		48.6 (32.3-66.9)	
Other		22.5 (7.1-41.3)		37.0 (17.0-82.3)		64.2 (34.9-87.1)	
Birth weight, kg	(N=154)	*r* = 0.08	0.35	*r* = 0.03	0.69	*r* = 0.09	0.24
< 2.5 kg		16.3 (7.5-39.2)	0.99	30.0 (15.8-53.3)	0.89	39.4 (32.6-79.3)	0.67
≥ 2.5 kg		18.9 (8.3-34.5)		34.0 (16.1-54.4)		50.7 (32.3-71.2)	
Birth length, cm	(N=150)	*r* = 0.09	0.28	*r* = 0.07	0.37	*r* = 0.08	0.32
Single ventricle			0.25		0.09		0.65
Yes		13.8 (6.9-27.7)		23.9 (13.7-38.7)		44.2 (34.9-63.4)	
No		19.2 (10.0-38.8)		34.4 (16.4-59.7)		51.5 (31.7-73.9)	
Pre-operative ventilation			0.64		0.55		0.17
Yes		16.7 (9.2-40.0)		25.7 (14.5-54.3)		45.8 (26.8-63.6)	
No		19.2 (8.3-34.3)		33.9 (16.4-54.4)		50.2 (34.9-75.4)	
On diuretics day prior to surgery			0.70		0.96		0.37
Yes		18.8 (10.0-34.5)		29.2 (16.1-53.3)		51.5 (35.4-73.9)	
No		18.8 (8.3-40.0)		34.0 (16.0-54.4)		49.2 (30.0-73.1)	
Pre-operative feeding			0.03		0.07		0.20
Yes		20.0 (10.0-40.0)		35.6 (16.4-60.3)		50.1 (34.9-75.4)	
No		13.1 (5.2-26.7)		24.0 (11.9-49.2)		47.7 (30.6-63.5)	

Age at surgery, days		*r* = 0.37	<.0001	*r* = 0.33	<.0001	*r* = 0.28	0.0004
< 30 days		12.5 (5.8-26.7)	<.0001	21.7 (12.5-44.7)	<.0001	45.2 (31.5-63.1)	0.001
≥ 30 days		30.9 (16.5-57.1)		44.4 (29.6-83.5)		66.7 (38.1-102)	
Weight at surgery, kg		*r* = 0.50	<.0001	*r* = 0.47	<.0001	*r* = 0.43	<.0001
Height at surgery, cm		*r* = 0.51	<.0001	*r* = 0.46	<.0001	*r* = 0.41	<.0001
BSA at surgery, m^2		*r* = 0.52	<.0001	*r* = 0.48	<.0001	*r* = 0.44	<.0001
STAT category			0.002		0.002		0.12
1 to 3		25.6 (15.0-52.3)		42.0 (24.6-79.3)		55.3 (34.5-97.3)	
4 or 5		13.8 (6.3-29.7)		24.5 (12.9-46.9)		48.1 (31.7-64.1)	
On inotropes at surgery			0.56		0.16		0.14
Yes		15.6 (7.5-37.5)		22.6 (15.8-50.0)		43.5 (32.3-54.7)	
No		19.2 (9.2-36.7)		34.1 (18.5-60.3)		52.6 (33.3-77.4)	
CPB time, minutes		*r* = 0.03	0.67	*r* = 0.08	0.34	*r* = 0.09	0.26
Cross-clamp time, minutes		*r* = 0.15	0.07	*r* = 0.14	0.09	*r* = 0.10	0.21
DHCA			0.10		0.02		0.37
Yes		13.8 (6.9-34.3)		22.2 (12.1-51.0)		45.2 (33.4-63.6)	
No		20.0 (11.0-39.6)		38.4 (19.9-60.0)		52.5 (31.9-76.4)	
DHCA time, minutes	(N=59)	*r* = -0.27	0.04	*r* = -0.31	0.02	*r* = -0.11	0.40

Abbreviations: BSA, body surface area; STAT, Society of Thoracic Surgeons-European Association for Cardio-Thoracic Surgery; CPB, cardiopulmonary bypass; DHCA, deep hypothermic circulatory arrest.

* Data are presented as Median (interquartile range) for categorical variables and *r*, Spearman correlation coefficient for continuous variables.

§ P-value from Wilcoxon rank sum test for categorical variables and Spearman correlation coefficient for continuous variables.
